# The Mediterranean-DASH Intervention for Neurodegenerative Delay (MIND) Diet and Metabolites in Chronic Kidney Disease

**DOI:** 10.3390/nu16152458

**Published:** 2024-07-29

**Authors:** Catharine A. Couch, Zsuzsanna Ament, Amit Patki, Naruchorn Kijpaisalratana, Varun Bhave, Alana C. Jones, Nicole D. Armstrong, Katharine L. Cheung, W. Taylor Kimberly, Hemant K. Tiwari, Marguerite Ryan Irvin

**Affiliations:** 1 Department of Epidemiology, School of Public Health, University of Alabama at Birmingham, Birmingham, AL 35294, USA; acjones@uab.edu (A.C.J.); nmda@uab.edu (N.D.A.); irvinr@uab.edu (M.R.I.); 2 Department of Neurology, Massachusetts General Hospital, Boston, MA 02114, USA; zament@mgh.harvard.edu (Z.A.); naruchorn.k@chula.ac.th (N.K.); wtkimberly@mgh.harvard.edu (W.T.K.); 3 Center for Genomic Medicine, Massachusetts General Hospital, Boston, MA 02114, USA; 4 Department of Biostatistics, School of Public Health, University of Alabama at Birmingham, Birmingham, AL 35233, USA; apatki@uab.edu (A.P.); htiwari@uab.edu (H.K.T.); 5 Division of Neurology, Department of Medicine and Division of Academic Affairs, Faculty of Medicine, Chulalongkorn University, Bangkok 10330, Thailand; 6 Harvard Medical School, Boston, MA 02115, USA; vbhave@hms.harvard.edu; 7 Division of Nephrology, Department of Medicine, Larner College of Medicine at the University of Vermont, Burlington, VT 05405-0068, USA; katharine.l.cheung@med.uvm.edu

**Keywords:** MIND diet, chronic kidney disease, metabolomics

## Abstract

The Mediterranean-DASH Intervention for Neurodegenerative Delay (MIND) is a hybrid of the Mediterranean and DASH (Dietary Approaches to Stop Hypertension) diets, and its association with renal outcomes remains unclear. In the REasons for Geographic and Racial Disparities in Stroke (REGARDS) cohort, diet data were collected at baseline using food frequency questionnaires. Modified Poisson regression was used to examine the association of MIND diet with incident chronic kidney disease (CKD). In the REGARDS stroke case-cohort, 357 metabolites were measured in baseline plasma. Weighted linear regression was used to test associations between MIND diet and metabolites. Weighted logistic regression was used to test associations between MIND-associated metabolites and incident CKD. Mediation analyses were conducted to determine whether metabolites mediated the relationship between MIND diet and CKD. A higher MIND diet score was associated with a decreased risk of incident CKD (risk ratio 0.90, 95% CI (0.86–0.94); *p* = 2.03 × 10^−7^). Fifty-seven metabolites were associated with MIND diet (*p* < 3 × 10^−4^). Guanosine was found to mediate the relationship between MIND diet and incident CKD (odds ratio for indirect effects 0.93, 95% CI (0.88–0.97); *p* < 0.05). These findings suggest a role of the MIND diet in renal outcomes.

## 1. Introduction

The Mediterranean-DASH Intervention for Neurodegenerative Delay (MIND) is a combination of the Mediterranean and Dietary Approaches to Stop Hypertension (DASH) diets and has been shown to be associated with slower cognitive decline [1] and reduced Alzheimer’s disease (AD) risk [2]. While it encompasses aspects of the Mediterranean and DASH diets, the MIND diet emphasizes foods and nutrients shown to be neuroprotective (i.e., green leafy vegetables, berries, nuts) [1,3,4]. Both the Mediterranean and DASH diets have been implicated in cardiometabolic disease, including cardiovascular disease [5,6], hypertension [7,8,9], stroke [10], and renal outcomes [11,12]. However, despite being a blend of these two diets, the relationship of the MIND diet with cardiometabolic disease, particularly renal outcomes, remains unclear.

With respect to renal outcomes, greater adherence to the Mediterranean diet has been associated with reduced serum creatinine and urea and increased creatinine clearance rate among healthy adults [12]. Similarly, findings from the Atherosclerosis Risk in Communities (ARIC) study found individuals with the lowest adherence to the DASH to be more likely to develop kidney disease than those with the highest adherence [11]. Less is known regarding the cardiometabolic implications of the MIND diet. There are many beneficial aspects of the MIND diet with respect to cardiometabolic disease, including low sodium and an emphasis on anti-inflammatory foods and nutrients [1]. These dietary characteristics have been associated with higher high-density lipoprotein cholesterol [13], lower blood pressure [7], lower inflammatory markers [14], and increased creatinine clearance rate [12]. Recent studies have shown a greater adherence to the MIND diet to be associated with a lower risk of cardiovascular events as well as a lower risk of all-cause death and cardiovascular death in patients with a preexisting atherosclerotic cardiovascular disease or history of stroke [15,16]. However, to our knowledge, no study has investigated the potential role of the MIND diet in chronic kidney disease (CKD).

A useful way of investigating pathways linking diet and disease is metabolomics [17,18]. Metabolomics is the analysis of small molecules within a biological sample and can capture unique information in healthy and pathophysiological states, as the metabolome is influenced by both genetics and environmental exposures [19]. Several metabolomics studies have identified metabolites and metabolomic pathways involved in the pathophysiology of CKD [20]. A recent study in the Reasons for Geographic and Racial Disparities in Stroke (REGARDS) cohort identified metabolites associated with a plant-based dietary pattern, the Mediterranean diet, and the DASH diet. Some of these diet-associated metabolites were found to mediate the relationship between the plant-based dietary pattern and stroke risk [17]. However, there is limited knowledge regarding metabolites associated with the MIND diet. Such knowledge could provide insight into molecular pathways linking the MIND diet and disease outcomes.

In the present study, we sought to test the hypothesis that high adherence to the MIND diet would be associated with a decreased risk of incident CKD and that this relationship would be mediated by specific metabolites. To answer this question, the objectives of this study were (1) to determine if the MIND diet is associated with incident CKD in the REGARDS cohort, (2) to identify metabolites that are associated with the MIND diet, and (3) to determine if these specific metabolites mediate the relationship between MIND diet and CKD in REGARDS.

## 2. Materials and Methods

### 2.1. Study Population

The REGARDS study was a national, population-based, longitudinal study whose primary focus was to identify racial and geographic disparities in stroke. A total of 30,239 non-Hispanic White and Black adults ≥ 45 years of age were enrolled between 2003 and 2007. The study oversampled individuals who were Black and residents of the southeastern United States. This is an area of the United States known as the stroke belt and includes Louisiana, Arkansas, Mississippi, Alabama, Tennessee, Georgia, North Carolina, and South Carolina, as well as the stroke “buckle” region along the coastal plains of North Carolina, South Carolina, and Georgia. Additional methods for enrollment and study design have been previously described [21,22]. At baseline, participants were contacted by phone, and consented individuals completed a telephone interview to obtain clinical, demographic, and lifestyle information. An in-home visit was conducted 2–3 weeks later, and blood samples were collected by venipuncture in EDTA plasma tubes. Blood samples were stored on ice until centrifuged and subsequent plasma aliquots were shipped overnight on ice and stored in a central laboratory at −80 °C [23]. Living participants or their proxies were followed up every 6 months by telephone for stroke and hospitalization ascertainment. Participants were invited to undergo a second in-person visit starting in April 2013 during which baseline procedures were repeated. The second in-person visits were completed in December 2016. A total of 16,150 participants returned for a second visit.

In the present analysis, the full REGARDS cohort was used for non-metabolomic analyses. For analyses involving metabolomics, we included participants who were part of the REGARDS stroke case-cohort nested within the full REGARDS cohort. The REGARDS stroke case-cohort includes all adjudicated stroke cases through 1 April 2019, and a randomly selected subset of participants from the full REGARDS cohort (i.e., cohort random sample), selected to be representative across age, sex, and race categories [24,25]. All participants provided written informed consent, and the study was approved by all participating institutional review boards.

### 2.2. MIND Diet

Diet intake was assessed at baseline with the Block 98 food frequency questionnaire (FFQ) (NutritionQuest, Berkeley, CA, USA) [26]. Participants were asked to recall their usual dietary intake for the past year. After the in-home visit, FFQs were self-administered by participants and mailed back to the REGARDS Operations Center. Completed FFQs were forwarded to NutritionQuest for processing and analysis of the mean daily intake (grams) of individual FFQ food items. Subjects with missing, incomplete, or implausible FFQ results were excluded from analyses [27].

MIND diet scores were obtained from FFQ results using previously described approaches [1,2]. Briefly, the MIND diet consists of 15 components. Ten of these components are considered brain healthy and include green leafy vegetables, other vegetables (e.g., peppers, carrots), berries, nuts, olive oil, whole grains, fish, beans, poultry, and wine. Five of these components are considered brain unhealthy and include butter, cheese, red meat and products, fast fried foods, and pastries and sweets. Each component is assigned a score of 0, 0.5, or 1 depending on the number of servings consumed each day or week. The MIND diet score is then calculated as the sum of all 15 components. The MIND diet score has a range from 0 to 15, with higher scores indicating greater adherence to the MIND diet.

### 2.3. Outcome Definition

Kidney function was measured at the baseline and second in-person visits as previously described [28]. Serum creatinine was measured using an isotope-dilution mass spectrometry-traceable method [29] with the Vitros 950IRC instrument (Johnson & Johnson Clinical Diagnostics, Raritan, NJ, USA). Serum cystatin C was measured with high-sensitivity particle-enhanced immunonephelometry (N Latex Cystatin C on the BNII; Dade Behring, Deerfield, IL, USA). Estimated glomerular filtration rate (eGFR) was calculated at baseline and follow-up as a function of age, sex, serum creatinine, and cystatin C using the 2021 CKD Epidemiology Collaboration (CKD-EPI) equation [30]. Incident CKD was defined as an eGFR <60 mL/min/1.73 m^2^ at follow-up and a ≥40% decrease in eGFR from baseline. We expanded this definition for incident CKD to an eGFR <60 mL/min/1.73 m^2^ at follow-up in secondary analyses. These results are in the Supplemental Material (Tables S1–S3).

### 2.4. Metabolomics

Metabolomics analyses were carried out in baseline samples as part of an ancillary study in the REGARDS stroke case-cohort [24,25]. EDTA plasma was shipped on dry ice to Massachusetts General Hospital. Targeted metabolomics of aqueous compounds (162 metabolites) was performed using dual Infinity II 1290 high-performance liquid chromatography pumps and a 6495 QQQ tandem mass spectrometer (Agilent, Santa Clara, CA, USA), as previously detailed [31,32]. The same instrumentation was used to measure lipid metabolites, which were extracted from 10 µL plasma aliquots with 190 µL of isopropanol containing 1,2-didodecanoyl-*sn*-glycero-3-phosphocholine as an internal standard (Avanti Polar Lipids, Alabaster, AL, USA) [33]. After centrifugation (10 min at 9000× *g*, at 4 °C), supernatants were injected directly onto a Kinetex 100 × 2.1 mm column (2.6 µm) (Phenomenex, Torrance, CA, USA). The mass spectrometer was tuned to profile a total of 195 plasma polar and nonpolar lipids. Lipids were denoted by headgroup and total carbon and double bond content. Mobile phase A was 0.1% formic acid and 10 mM ammonium formate in water. Mobile phase B was 0.1% formic acid in acetonitrile: isopropanol (10:90 *v*:*v*). The gradient increased from 30% to 99% B over 17 min at a flow rate of 0.35 mL/min. A total of 357 metabolites were detected. Due to the non-normal distribution of metabolite levels, all values were rank-based inverse normal transformed before statistical analyses.

### 2.5. Covariates

Demographic characteristics included self-reported age, race (Black or White), sex (male or female), education, income, and region. Education levels were grouped as less than high school, high school graduate, some college, or college graduate and above. Income levels were grouped as <USD 20,000, from USD 20,000 to USD 34,999, from USD 35,000 to USD 74,999, ≥USD 75,000, or refused. The region was grouped as stroke belt/buckle or non-belt/buckle. Lifestyle characteristics included smoking status, alcohol consumption, and exercise. Smoking status was grouped as current vs. never/past. Alcohol consumption was grouped as none, moderate, or heavy. Exercise levels were grouped as none vs. some. Clinical characteristics included body mass index (BMI), systolic blood pressure (SBP), hypertension, diabetes, and albumin creatinine ratio (ACR). BMI was calculated as weight in kilograms divided by height in meters squared (kg/m^2^). Hypertension was defined as self-reported hypertension, use of antihypertensive medication, or by the 2017 American College of Cardiology/American Heart Association guidelines (SBP ≥ 130 mm Hg or diastolic blood pressure (DBP) ≥ 80 mm Hg) [34]. Diabetes was defined as fasting plasma glucose of ≥126 mg/dL and/or non-fasting plasma glucose of ≥200 mg/dL or the use of insulin or oral diabetes medication. Urine albumin was measured by nephelometry using the BNII ProSpec nephelometer (now Siemens AG), and urine creatinine was measured using the Modular-P chemistry analyzer (Roche/Hitachi, www.roche.com). ACR was calculated in milligrams per gram.

### 2.6. Statistical Analyses

**Figure 1:**
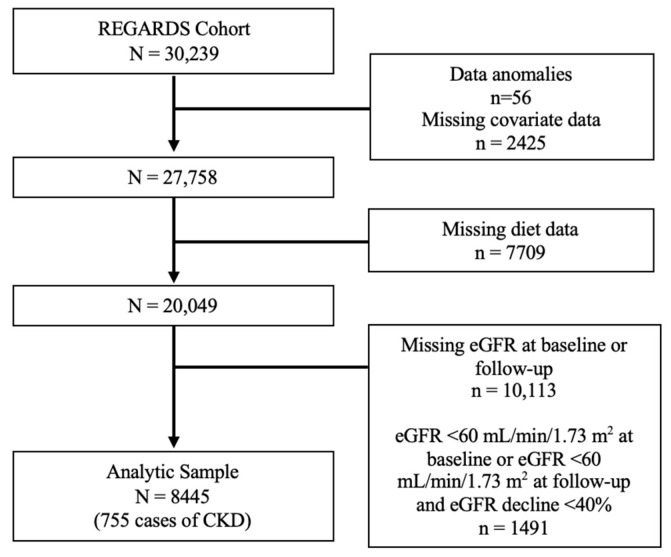
Participant flow chart in the REGARDS cohort. Abbreviations: CKD, chronic kidney disease; eGFR, estimated glomerular filtration rate.

For the primary analyses carried out in the full REGARDS cohort, participants were excluded if they were missing eGFR measures at baseline or follow-up, had a baseline eGFR <60 mL/min/1.73 m^2^, had new eGFR < 60 at follow-up but an eGFR decline <40% at follow-up, or were missing any covariate or diet data (Figure 1). Modified Poisson regression was used to assess the relationship between MIND diet score as a continuous predictor and incident CKD. The base model adjusted for age, sex, race, and total calories. Model 2 was further adjusted for BMI, diabetes, and hypertension. Model 3 additionally included alcohol intake, smoking, physical activity, education, income, and region as covariates. A *p*-value of <0.05 was considered statistically significant.

Analyses investigating the association of the MIND diet with metabolites were restricted to participants with complete diet and metabolomic data (i.e., if participants were missing CKD data, they were still included in this analytic sample; Figure S1). Weighted linear regression models were used to evaluate the association between the MIND diet score as a continuous predictor and 357 metabolites. The base model adjusted for age, race, sex, and total calories. The fully adjusted model additionally included alcohol, smoking, and BMI. An adjusted *p*-value of <3 × 10^−4^ (176 effective tests) was used to account for multiple tests using the Li and Ji method. The Li and Ji method is less conservative than the Bonferroni correction and is able to account for correlations among individual metabolites [35]. Sensitivity analyses were conducted in the cohort random sample with the exclusion of stroke cases.

Analyses investigating the association of metabolites with incident CKD were restricted to participants with complete metabolomic and CKD data (i.e., if participants were missing diet data, they were still included in this analytic sample; Figure S1). Weighted logistic regression models were used to assess the relationship between MIND-associated metabolites and incident CKD. The base model adjusted for age, race, and sex. The fully adjusted model additionally adjusted for alcohol, smoking, and BMI. Metabolites with a *p*-value < 2 × 10^−3^ (adjusted for multiple testing, 25 effective tests) were carried forward for mediation analyses [35]. Sensitivity analyses were conducted in the cohort random sample with the exclusion stroke cases.

Mediation analyses between MIND diet, metabolites, and incident CKD were conducted in SAS using Proc CAUSALMED. The analytic sample for mediation analyses included participants with complete diet, metabolomic, and CKD data (Figure S1). The adjusted model included age, race, and sex. A *p*-value of <0.05 was considered statistically significant. All analyses were run in SAS version 9.4 and R version 4.2.2.

## 3. Results

### 3.1. Characteristics of Participants

In the full REGARDS cohort, a total of 8445 participants had complete diet, CKD, and covariate data (Figure 1). The mean age was 62.2 ± 8.01 years, 58.4% were female, and 29.6% were Black. The mean MIND diet score was 7.4 ± 1.8, with a range from 2 to 13.5, and there were 755 cases of incident CKD. Participant characteristics are displayed in Table 1 by quartile of the MIND diet score. When compared to participants in the lowest MIND diet quartile, participants in the highest MIND diet quartile tended to be female, White race, have higher educational attainment, have a higher income, reside in a non-belt/buckle region, not smoke, and be physically active. They were also less likely to have diabetes and hypertension.

### 3.2. The MIND Diet and CKD

Table 2 displays risk ratios (RR) for models investigating the association between MIND diet score and incident CKD. In the fully adjusted model, there was a 10% lower risk of incident CKD per one unit increase in MIND diet score (RR = 0.90, 95% CI: 0.86, 0.94; *p* = 2.03 × 10^−7^). Results were comparable when the expanded definition of CKD was considered (Table S1).

### 3.3. Metabolites Associated with the MIND Diet

**Figure 2:**
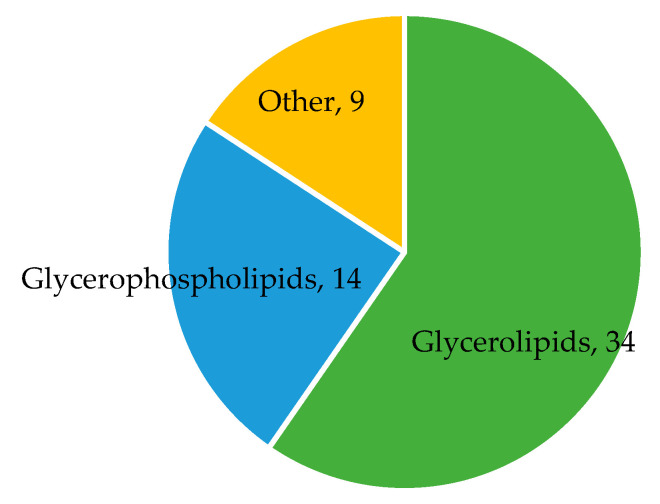
Metabolite classes associated with the MIND diet. The diagram displays the number of metabolites in each metabolite class found to be associated with the MIND diet. Sample size N = 1451. Metabolites in other classes include C7 carnitine, pantothenic acid, gluconic acid, sphingolipid SMd(18:1/25:0), uric acid, guanosine, S-adenosyl-L-homocysteine, dimethylguanidino valeric acid, and indole-3-propionic acid.

In the REGARDS case-cohort, 1451 participants had complete diet and metabolomic data (Figure S1). A total of 57 metabolites were associated with the MIND diet (*p* < 3 × 10^−4^; Figure 2, Table S4). These included 14 glycerophospholipids, 34 glycerolipids, 1 sphingolipid (SMd(18:1/25:0)), 1 acyl carnitine (C7 carnitine), 1 vitamin (pantothenic acid), 1 carbohydrate (gluconic acid), 1 indole (indole-3-propionic acid, IPA), 1 keto acid (dimethylguanidino valeric acid, DMGV), 1 lactone (S-adenosyl-L-homocysteine, SAH), 1 purine nucleoside (guanosine), and 1 purine (uric acid). Results were consistent when analyses were restricted to non-cases in the cohort random sample (Table S5).

### 3.4. MIND-Metabolites Associated with CKD

**Figure 3:**
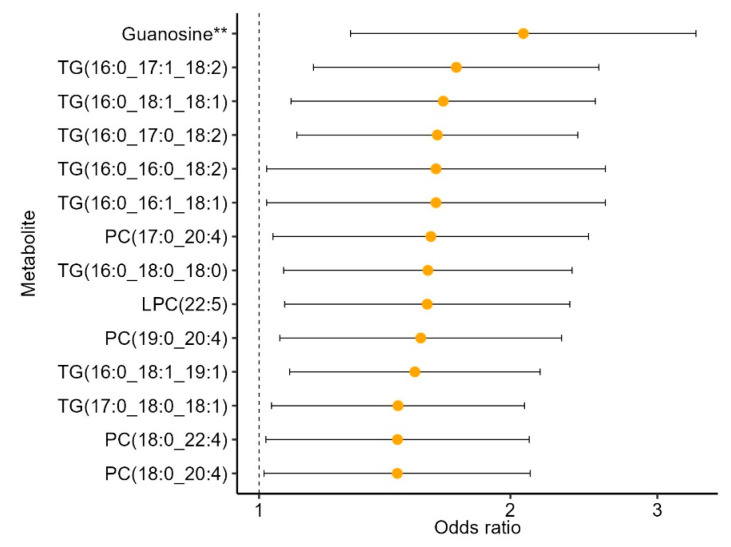
MIND metabolites associated with incident CKD in the REGARDS case-cohort ancillary study. All 14 metabolites were inversely associated with the MIND diet. Models adjusted for age, sex, race, alcohol intake, smoking, and BMI. ** indicates *p* < 2 × 10^−3^ (adjusted for multiple testing). Sample size N = 628. Abbreviations: TG, triacylglycerol, PC, phosphatidylcholine; LPC, lysophosphatidylcholine.

The 57 metabolites found to be associated with the MIND diet were carried forward to examine associations with incident CKD. These analyses were restricted to participants with complete metabolite and CKD data (N = 628, Figure S1). Of the 57 metabolites associated with the MIND diet, 14 met nominal significance with incident CKD (*p* < 0.05, Figure 3). All 14 of these metabolites were inversely associated with the MIND diet and associated with increased odds of CKD. Guanosine was the only metabolite to meet the multiple testing correction *p*-value of <2 × 10^−3^, with higher levels of guanosine associated with greater odds of incident CKD. These results were comparable when the expanded definition of CKD was considered in secondary analyses (19 metabolites associated with CKD, eGFR < 60 mL/min/1.73 m2, Table S2). Results from sensitivity analyses were also consistent when restricted to non-cases in the cohort random sample (Table S6).

### 3.5. Mediation Analysis

As guanosine was the only metabolite meeting the adjusted *p*-value, it was carried forward for mediation analysis to explore its potential as a mediator linking the MIND diet to CKD. Mediation analyses were restricted to participants with complete diet, metabolomic, and CKD data (N = 474, Figure S1). In both the unadjusted and adjusted models, guanosine was found to partially mediate the relationship between the MIND diet and incident CKD (*p* < 0.05 for indirect effects, Table 3), suggesting that guanosine partially removes the protective effect of the MIND diet.

## 4. Discussion

In the present study, we investigated the relationship of the MIND diet with incident CKD in the REGARDS cohort, while also leveraging metabolomic data from a REGARDS ancillary study to identify metabolites potentially mediating this relationship. Greater adherence to the MIND diet was associated with a decreased risk of incident CKD. One metabolite, guanosine, partially mediated the association between the MIND diet and CKD. These findings may suggest a potential role for the MIND diet in renal outcomes.

High adherence to the MIND diet was associated with a decreased risk of CKD. The Mediterranean and DASH diets have been previously associated with renal outcomes [11,12], but to our knowledge, this is the first study demonstrating an association between the MIND diet and CKD. The MIND diet has many beneficial aspects in the context of kidney function and disease, including low sodium [1,2,11,36], an emphasis on anti-inflammatory foods and nutrients (i.e., berries, leafy greens) [1,2,37,38,39], and low intake of ultra-processed foods [1,2,40].

Metabolomic analyses indicated 57 metabolites to be associated with the MIND diet. Over half of these metabolites were lipids, belonging to the glycerophospholipid or glycerolipid classes. Many of the lipid metabolites positively associated with the MIND diet were highly unsaturated, containing one or more chain(s) of eicosapentaenoic acid (EPA), docosahexaenoic acid (DHA), and/or docosapentaenoic acid (DPA). Highly unsaturated lipid metabolites have previously been associated with the Mediterranean diet and several of the food groups making up the Mediterranean diet (i.e., high intakes of olive oil, nuts, fish/seafood, and low intakes of sweets and sugar-sweetened beverages) [18]. Many of these foods are also emphasized in the MIND diet and, therefore, it is not surprising we observed similar associations. Other metabolites found to be positively associated with the MIND diet included pantothenic acid and indole-3-propionic acid (IPA). Pantothenic acid, also known as vitamin B5, is an essential nutrient found widely in numerous foods [41]. IPA is a tryptophan metabolite, and high levels have been associated with diets high in polyphenols, which are natural compounds found in fruits, vegetables, green tea, and coffee [42]. Our findings are consistent with a previous REGARDS study which found IPA to be positively associated with the Mediterranean and DASH diets [17].

Metabolites found to be inversely associated with the MIND diet included S-adenosyl-L-homocysteine (SAH), dimethylguanidino valeric acid (DMGV), gluconic acid, guanosine, and uric acid. SAH is the immediate precursor to homocysteine and diets low in fruits and vegetables have been associated with higher homocysteine levels [43]. DMGV is a metabolite formed from the transamination of asymmetric dimethylarginine and has been shown to be associated with a high intake of sugary beverages and low intakes of fruits and vegetables [44]. As a byproduct of glucose oxidation, gluconic acid occurs naturally in many foods [45]. Guanosine is a purine nucleoside that is broken down into uric acid, which is the end product of purine metabolism [46]. Consistent with our findings, prior studies have shown guanosine and uric acid to be inversely associated with the Mediterranean and DASH diets [17,47,48] and positively associated with high consumption of animal products, fried foods, and sugary beverages [17,49].

Of the 57 metabolites associated with the MIND diet, 14 were associated with incident CKD. These included eight triacylglycerols, four phosphatidylcholines, one lysophosphatidylcholine, and guanosine. Higher levels of all these metabolites were associated with a lower MIND diet score (indicating low adherence to the MIND diet) and increased odds for incident CKD. Guanosine was the only metabolite found to be inversely associated with CKD and to partially mediate the relationship between the MIND diet and CKD. As a purine nucleoside, guanosine is cleared from the plasma via glomerular filtration, likely explaining the association with reduced eGFR [50]. The observed mediation effect of guanosine on the relationship between the MIND diet and incident CKD may suggest that guanosine metabolism is implicated in decreasing the protective effect of the MIND diet on CKD. However, it is still possible that this effect is due to the combined but independent effects of diet (i.e., consumption of foods with high purine nucleoside content) and disease (i.e., reduced renal clearance of guanosine and/or dysregulation of purine metabolism due to the pathophysiology of the disease itself). Nonetheless, these findings will need to be replicated and warrant further investigation.

Strengths of the present study include the use of eGFR to define incident CKD, the large sample size, and the targeted mass spectrometry platform for metabolomics data [32]. There are also limitations. First and foremost, the observational study design limited our ability to draw conclusions regarding potential causal relationships between the MIND diet, metabolites, and disease outcomes. Additionally, the lack of a replication cohort limits our ability to extend these findings to other populations. Finally, the REGARDS study is an older cohort with an average age of 68 years, and these findings may not be generalizable to younger, healthier populations.

In conclusion, a low adherence to the MIND diet is associated with incident CKD in the REGARDS cohort, and guanosine mediates this relationship. Our findings suggest that the MIND diet may have benefits in renal disease, and that guanosine metabolism may be implicated in these effects. Follow-up studies will need to confirm these findings and explore the potential of the MIND diet for the prevention and management of renal disease.

## Figures and Tables

**Table 1:** Baseline characteristics of REGARDS participants by quartile of MIND diet score.

Characteristic	Q1 (Lowest Consumption) (N = 2112)	Q2 (N = 2111)	Q3 (N = 2111)	Q4 (Greatest Consumption) (N = 2111)
**MIND diet score**	5.08 ± 0.8	6.7 ± 0.4	8.0 ± 0.4	9.8 ± 0.8
**Age, years**	61.4 ± 8.1	62.0 ± 8.0	62.4 ± 7.9	63.0 ± 7.9
**Female sex**	1063 (50.3)	1168 (55.3)	1288 (61.0)	1411 (66.8)
**Black race**	774 (36.7)	653 (30.9)	576 (27.3)	500 (23.7)
**Education**				
• Less than HS	212 (10.0)	113 (5.4)	87 (4.1)	78 (3.7)
• HS graduate	644 (30.5)	495 (23.5)	405 (19.2)	342 (16.2)
• Some college	599 (28.4)	583 (27.6)	573 (27.1)	482 (22.8)
• College graduate and above	657 (31.1)	920 (43.6)	1046 (49.6)	1209 (57.3)
**Income**				
• <USD 20,000	323 (15.3)	235 (11.1)	193 (9.1)	144 (6.8)
• USD 20,000–USD 34,999	522 (24.7)	464 (22.0)	410 (19.4)	388 (18.4)
• USD 35,000–USD 74,999	698 (33.1)	720 (34.1)	755 (35.8)	730 (34.6)
• ≥USD 75,000	364 (17.2)	481 (22.8)	532 (25.2)	616 (29.2)
• Refused	205 (9.7)	211 (10.0)	221 (10.5)	233 (11.0)
**Region**				
• Belt/buckle	1296 (61.4)	1257 (59.6)	1144 (54.2)	1061 (50.3)
• Non-belt/buckle	816 (38.6)	854 (40.5)	967 (45.8)	1050 (49.7)
**Alcohol Use**				
• None	1308 (61.9)	1179 (55.9)	1099 (52.1)	992 (47.0)
• Moderate	701 (33.2)	833 (39.5)	900 (42.6)	1006 (47.7)
• Heavy	103 (4.9)	99 (4.7)	112 (5.3)	113 (5.4)
**Current Smoker**	367 (17.4)	245 (11.6)	205 (9.7)	105 (5.0)
**Physical Activity**				
• None	745 (35.3)	629 (29.8)	534 (25.3)	428 (20.3)
• Some	1367 (64.7)	1482 (70.2)	1577 (74.7)	1683 (79.7)
**BMI**, kg/m^2^	29.7 ± 6.0	29.2 ± 5.8	28.7 ± 5.7	27.7 ± 5.6
**Diabetes**	339 (16.1)	317 (15.0)	251 (11.9)	226 (10.7)
**Hypertension**	1486 (70.4)	1425 (67.5)	1373 (65.0)	1283 (60.8)
**eGFR**, mL/min/1.73 m^2^	100.9 ± 21.1	102.4 ± 21.9	104.1 ± 21.6	106.4 ± 26.2
**ACR**, mg/g	6.2 [4.0–11.1]	6.2 [4.2–11.0]	6.3 [4.3–10.6]	6.2 [4.3–10.7]

Categorical variables are expressed as N (%) and continuous variables are expressed as mean ± SD or median [interquartile range]. Abbreviations: HS, high school; BMI, body mass index; eGFR, estimated glomerular filtration rate; ACR, albumin creatinine ratio.

**Table 2:** Association of the MIND diet with incident CKD in the REGARDS cohort.

Model	RR (95% CI)	*p*-Value
Model 1	0.85 (0.82, 0.88)	4.46 × 10^−18^
Model 2	0.87 (0.84, 0.90)	4.74 × 10^−13^
Model 3	0.90 (0.86, 0.94)	2.03 × 10^−7^

Model 1 adjusts for age, sex, race, and total calories. Model 2 further adjusts for BMI, diabetes, and hypertension. Model 3 further adjusts for alcohol intake, smoking, physical activity, education, income, and region. RRs can be interpreted as the change in CKD risk per one unit increase in MIND diet score. Sample size N = 8445. Abbreviations: RR, risk ratio; CI, confidence interval.

**Table 3:** Guanosine mediates the relationship between the MIND diet and incident CKD.

	OR Total Effect	OR Natural Direct Effect	OR Natural Indirect Effect	*p*-Value For Indirect Effect
Model 1	0.87 [0.74, 1.00]	0.94 [0.79, 1.08]	0.93 [0.89, 0.97]	0.001
Model 2	0.86 [0.72, 1.00]	0.93 [0.78, 1.08]	0.93 [0.88, 0.97]	0.002

Model 1 is unadjusted. Model 2 adjusted for age, race, and sex. Sample size N = 474. Abbreviations: OR, odds ratio; CKD, chronic kidney disease.

## Data Availability

The REGARDS study database contains identifying participant information and therefore cannot be made publicly available. Data supporting the findings of this study are available on reasonable request to researchers trained in human subject confidentiality protocols from the REGARDS executive committee.

## Supplementary Materials

The following supporting information can be downloaded at: https://www.mdpi.com/article/10.3390/nu16152458/s1, Figure S1: Participant flow chart in the REGARDS case-cohort ancillary study (analyses involving metabolite data); Table S1: Association of the MIND diet with incident CKD (eGFR < 60 mL/min/1.73 m^2^) in the REGARDS cohort; Table S2: MIND-metabolites associated with incident CKD (eGFR < 60 mL/min/1.73 m^2^) in the REGARDS case-cohort ancillary study; Table S3: Guanosine mediates the relationship between the MIND diet and incident CKD (eGFR < 60 mL/min/1.73 m^2^) in the REGARDS case-cohort ancillary study; Table S4: Metabolites associated with the MIND diet in the REGARDS case-cohort ancillary study; Table S5: Metabolites associated with the MIND diet in the REGARDS cohort random sample (sensitivity analyses); Table S6: MIND-metabolites associated with incident CKD in the REGARDS cohort random sample (sensitivity analyses).
