# Supplementary material for: The Mediterranean-DASH Intervention for Neurodegenerative Delay (MIND) Diet and Metabolites in Chronic Kidney Disease
Source: Nutrients. 2024 Jul 29;16(15):2458. doi: 10.3390/nu16152458 (PMC11314466; doi:10.3390/nu16152458)

Figure S1. Participant flow chart in the REGARDS case-cohort ancillary study (analyses involving metabolite data).

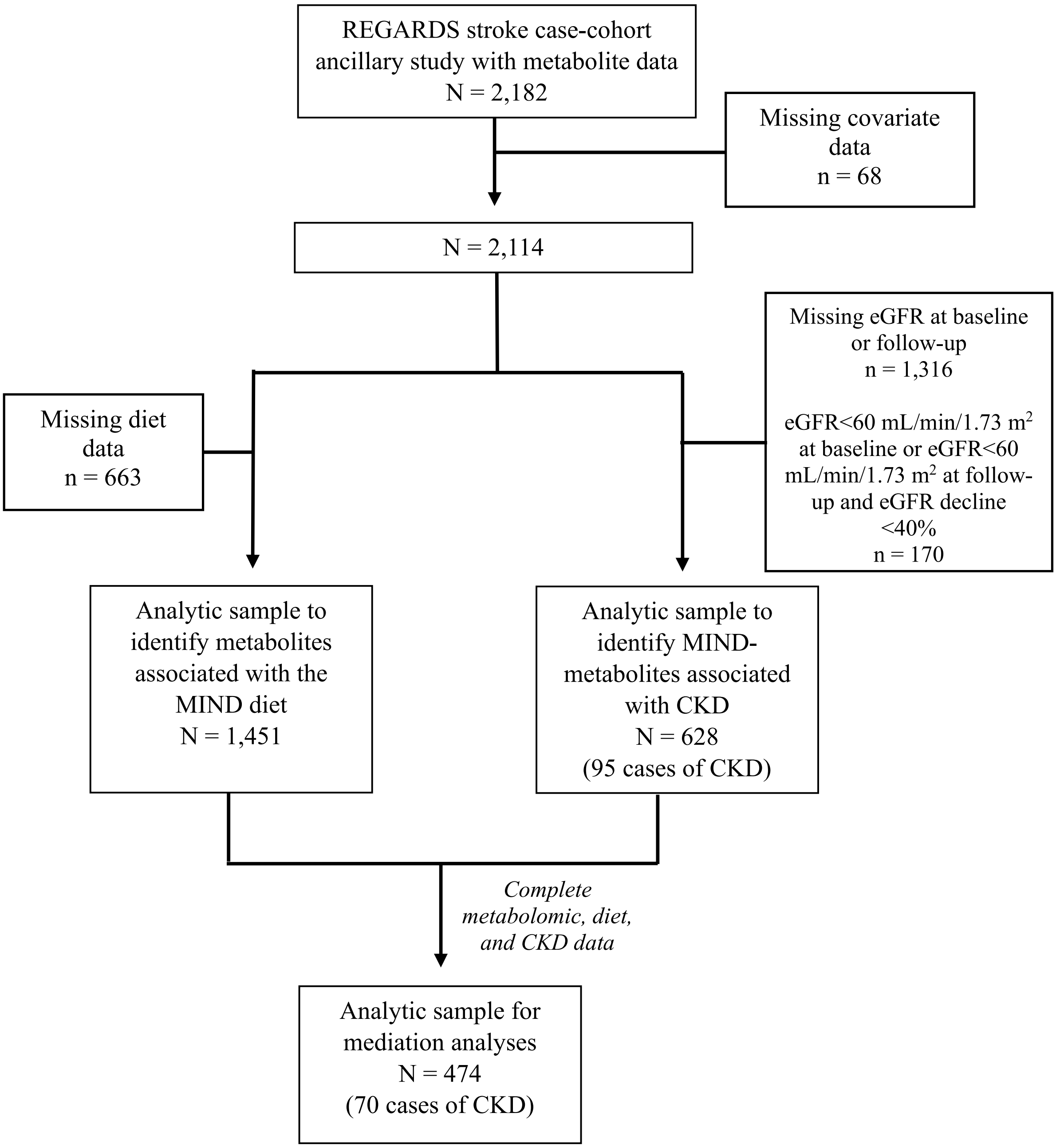

Supplement: Supplementary file 1 [file nutrients-16-02458-s001.zip › Supplemental Figures 070824.pdf]
